# The nuclear proteome of *Trypanosoma brucei*

**DOI:** 10.1371/journal.pone.0181884

**Published:** 2017-07-20

**Authors:** Carina Goos, Mario Dejung, Christian J. Janzen, Falk Butter, Susanne Kramer

**Affiliations:** 1 Department of Cell and Developmental Biology, Biocenter, University of Würzburg, Am Hubland, Würzburg, Germany; 2 Institute of Molecular Biology (IMB), Ackermannweg 4, Mainz, Germany; Centro de Investigacion y de Estudios Avanzados del Instituto Politecnico Nacional, MEXICO

## Abstract

*Trypanosoma brucei* is a protozoan flagellate that is transmitted by tsetse flies into the mammalian bloodstream. The parasite has a huge impact on human health both directly by causing African sleeping sickness and indirectly, by infecting domestic cattle. The biology of trypanosomes involves some highly unusual, nuclear-localised processes. These include polycistronic transcription without classical promoters initiated from regions defined by histone variants, trans-splicing of all transcripts to the exon of a spliced leader RNA, transcription of some very abundant proteins by RNA polymerase I and antigenic variation, a switch in expression of the cell surface protein variants that allows the parasite to resist the immune system of its mammalian host. Here, we provide the nuclear proteome of procyclic *Trypanosoma brucei*, the stage that resides within the tsetse fly midgut. We have performed quantitative label-free mass spectrometry to score 764 significantly nuclear enriched proteins in comparison to whole cell lysates. A comparison with proteomes of several experimentally characterised nuclear and non-nuclear structures and pathways confirmed the high quality of the dataset: the proteome contains about 80% of all nuclear proteins and less than 2% false positives. Using motif enrichment, we found the amino acid sequence KRxR present in a large number of nuclear proteins. KRxR is a sub-motif of a classical eukaryotic monopartite nuclear localisation signal and could be responsible for nuclear localization of proteins in *Kinetoplastida* species. As a proof of principle, we have confirmed the nuclear localisation of six proteins with previously unknown localisation by expressing eYFP fusion proteins. While proteome data of several *T*. *brucei* organelles have been published, our nuclear proteome closes an important gap in knowledge to study trypanosome biology, in particular nuclear-related processes.

## Introduction

*Trypanosoma brucei* is a protozoan, parasitic flagellate with a digenic life cycle that involves a mammalian host and the tsetse fly insect vector. The parasite causes African sleeping sickness as well as the related cattle disease Nagana and thus has a huge impact on human health. Mainly affected are rural areas of sub-Saharan Africa; some of these belong to the poorest regions in the world. Sleeping sickness is fatal if untreated and currently available drugs, in particular against the late stages of the disease, are difficult to administer and extremely toxic. Trypanosomes separated early in the eukaryotic lineage and evolved some interesting and in some cases unique biological mechanisms. Many of these are in fact located in the nucleus. For example the full reliance of the parasites on polycistronic transcription: tens to hundreds of functionally unrelated genes are co-transcribed together and subsequently processed by the addition of the intron of the spliced leader RNA in a trans-splicing reaction, which is coupled to polyadenylation of the upstream gene [1].

Trypanosome research has been eased by the availability of a large number of proteomic data. These are in particular important, since RNA and protein data poorly correlate, due to the absence of transcriptional control. Proteomic studies have analysed the proteomes of the different life cycle stages [2–4], changes during developmental differentiation [5] and the parasite’s phosphoproteome [6,7]. Additionally, different subcellular proteomes are available, for example the proteome of the flagellum [8,9], the nuclear pores [10], the mitochondrion [11] the cell surface [12] the mitochondrial importome [13] and the glysosome [14]; the later even for different life cycle stages [15].

The nuclear proteome is still missing and we set out to fill the gap. We performed label-free quantitative mass spectrometry of purified trypanosome nuclei and compared the protein enrichment against whole cell lysates identifying 764 proteins significantly enriched in purified nuclei. A comparison with the proteomes of known nuclear and non-nuclear structures allowed us to estimate the number of false positive proteins to be less than 2% and the completeness of the proteome to be about 80%. We found the motif KRxR, which is reminiscent of a nuclear localisation signal (NLS), significantly enriched within our nuclear proteome.

## Material and methods

### Trypanosomes

*Trypanosoma brucei* Lister 427 procyclic cells were used throughout. All experiments were performed with logarithmically growing trypanosomes at a cell density of less than 1•10^7^ cells/ml. The generation of transgenic trypanosomes was done using standard methods [49].

### Purification of trypanosome nuclei

The purification protocol was based on the purification of trypanosome nuclei described in [10]. For each purification, approximately 1•10^10^ procyclic cells at about 6•10^6^ cells/ml were cultivated in conical glass flasks (5 l volume) with gentle shaking. Cells were pelleted (1,700g, 10 min, 27°C) (swing out rotor 11650, Sigma 6-16K) and washed twice with SDM79 without serum and heme. From now work was done on ice. Cells were resuspended in 20 ml lysis buffer [10] and disrupted by a POLYTRON® homogenizer (PT 1200E, PT-DA 12/2 EC-E123, Kinematica AG, Switzerland) for at least 5 minutes at ^2^/_3_ of its maximum speed. Cell lysis was monitored by phase contrast and fluorescence microscopy, using DAPI staining for the detection of nuclei and kinetoplasts; part of this sample was kept for mass spectrometry (whole cell lysate, WCL). The cell lysate was underlaid with 10 ml underlay buffer [10] in a 30 ml COREX (No. 8445) glass tube and centrifuged (10,500g, 20 min, 4°C, rotor HB-6 in a Sorvall R6 plus centrifuge). The supernatant (containing mainly crude cytosol) was decanted and discarded. The pellet was immediately resuspended in 8 ml resuspension buffer [10], followed by further homogenisation with the POLYTRON® (5 min, ^2^/_3_ of maximum speed) and loaded on a three-step sucrose gradient (8 ml 2.01 M / 8 ml 2.1 M / 8 ml 2.3 M) in a Sorvall AH629 rotor tube (PA, thinwall, 38.5 ml, No 253050). After ultracentrifugation (25,000 rpm, 3.5 h, 4°C, Beckmann L7 centrifuge), the gradient was harvested from the top. The ring-shaped pellet at the bottom of the tube was resuspended in 2 ml 2.3 M sucrose. Samples were stained with DAPI and analysed microscopically. The pellet fraction contained the highest concentration in nuclei and the lowest concentration in visible contaminants and was subsequently used for mass spectrometry.

### Mass spectrometry

600 μl methanol, 150 μl chloroform and 450 μl water were added stepwise (with vigorous vortexing after each step) to 200 μl (10%) of the pellet fraction or 100 μl of the whole cell lysate. After centrifugation (5 min, 20,000 g), the upper, aqueous phase was discarded, and another 650 μl methanol was added (mixing by inversion). Proteins were pelleted by centrifugation (5 min, max. speed), resuspended in 100 μl 1 x NuPAGE LDS sample buffer (Thermo Fisher Scientific) with 100 mM DTT and incubated at 70°C for 10 minutes. Afterwards the samples were sonicated with the Bioruptor^®^ Plus sonication device (Diagenode, Belgium) (settings: high, 10 cycles, 30 sec ON /30 sec OFF).

The samples were in-gel digested and MS measurement was performed as previously described [50] with the following adaptations: the measurement time per sample was extended to 240 min. The four replicates were analysed with MaxQuant version 1.5.0.25 [51] with standard settings except LFQ quantitation and match between runs was activated. The trypanosome protein database TREU927 version 8.0 (11,567 entries) was downloaded from www.tritrypdb.org [18]. Filtering for proteins only identified by site, potential contaminants and reverse entries where conducted with custom R scripts. A second filter step is removing all protein groups with no unique and less than two peptides. Also the protein needs to be quantified in at least two samples in either NUC or WCL. Prior to imputation of missing LFQ values with a beta distribution ranging from 0.1 to 0.2 percentile within each sample, the values were log_2_ transformed. The mass spectrometry proteomics data have been deposited to the ProteomeXchange Consortium via the PRIDE [52] partner repository with the dataset identifier PXD006745".

### Expression of eYFP fusion proteins

All eYFP-fusion proteins (C-terminal tagging) were expressed from the endogenous locus, using the plasmid pPOTv4 as PCR template, exactly as described in [43].

### SDS page and Western blots

Proteins were separated on a 12% acrylamide gel. Western blots were performed according to standard protocols. The histone H3 antibody is described in [53].

### Microscopy

For microscopy, cells were washed in SDM79 without serum and heme and fixed at a density of less than 1•10^7^ cells/ml with 2.5% paraformaldehyde overnight at 4°C in suspension, washed twice in PBS and stained with DAPI. Z-stack images (60 stacks at 100 nm distance) were taken with a custom build TILL Photonics iMic microscope equipped with a sensicam camera (PCO), deconvolved using Huygens Essential software (Scientific Volume Imaging B. V., Hilversum, The Netherlands) and are presented as z-stack projections or single plane images. eYFP was monitored with the FRET-CFP/YFP-B-000 filter and DNA with the DAPI filter (Chroma Technology CORP, Bellows Falls, VT).

## Results and discussion

### Purification of trypanosome nuclei

**Fig 1:**
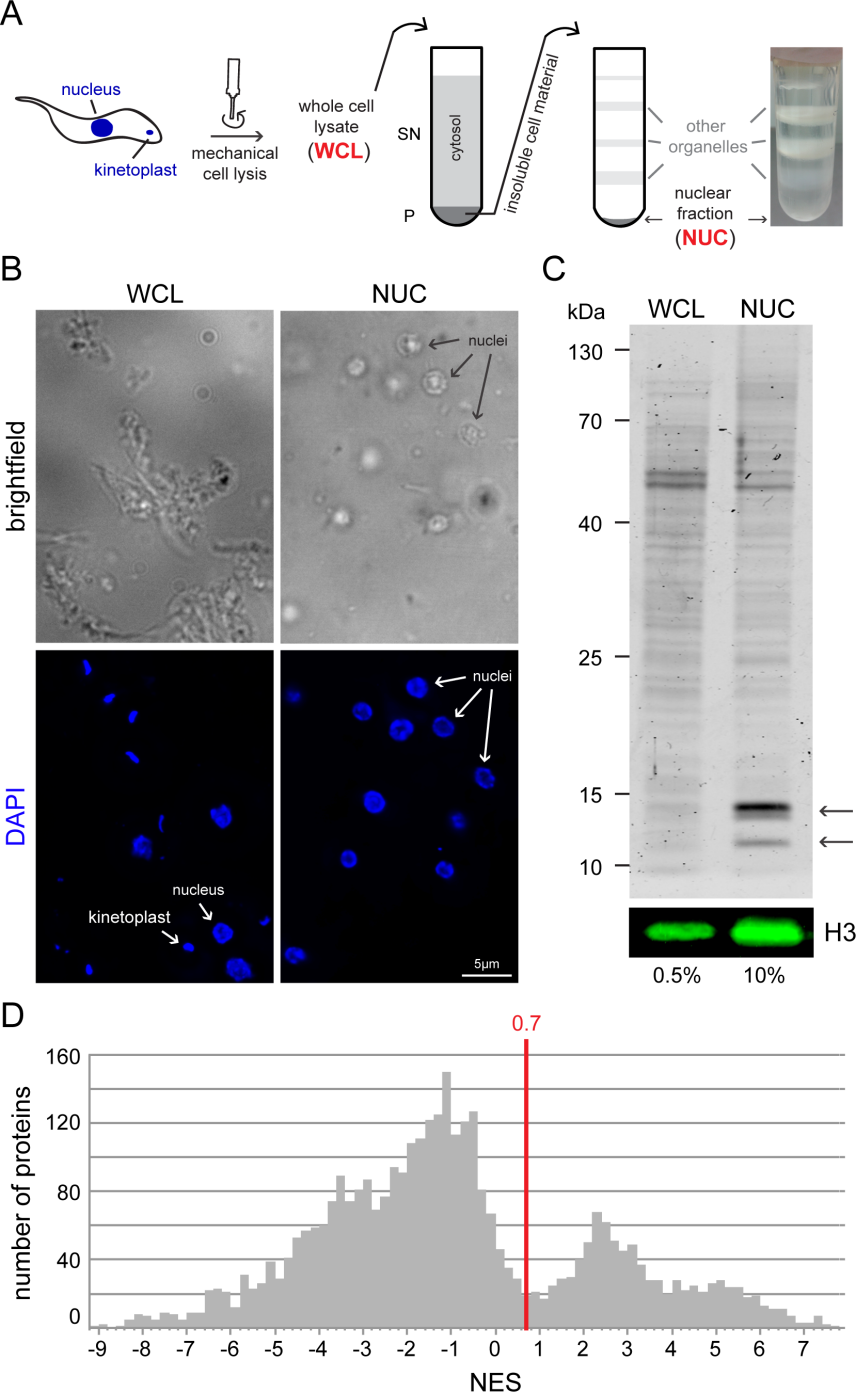
Purification of trypanosome nuclei. **A)** Schematics of the procedure. 1•10^10^ procyclic trypanosome cells were mechanically lysed with a POLYTRON® homogenizer (whole cell lysate, WCL). The insoluble cell fraction which includes the nuclei was separated from the soluble fraction via a sucrose cushion and further separated on a discontinuous sucrose gradient. Various organelles and cell fragments accumulate at the interfaces of the sucrose layers and are thus separated from the nuclei, which are found in the pellet fraction (NUC). A typical picture of an ultracentrifugation tube after centrifugation is shown on the right. **B)** Samples of whole cell lysates (WCL) and the nuclear fraction (NUC) were stained with DAPI and microscopically analysed. In the NUC sample, isolated nuclei are clearly visible as ovoids and few other structures are present, such as remnants of flagella (brightfield image). Nuclei are intact (native shape, nucleolus is visible by absence of DAPI staining) and only few kinetoplasts are visible (DAPI image). In contrast, the WCL sample contains remnants of whole cells, including both nuclei and kinetoplasts. Note that the samples were not fixed to the slide and moved during imaging; the different channels do not completely overlap. The DAPI image is shown as deconvolved z-stack projections, the brightfield image is a single plane. **C)** Enrichment in histones in fraction NUC. Coomassie-stained gel loaded with 0.5% of the WCL fraction and 10% of the NUC fractions (upper panel). The arrows point to the bands corresponding to histones. In addition, histone H3 was detected by western blot (lower panel, H3). **D)** NES histogram: For each 0.2 NES range, the number of proteins is shown. The NES of 0.7 that was used in this work to define a nuclear protein is shown as a red line.

Nuclei of procyclic *Trypanosoma brucei* Lister 427 cells were purified in four independent experiments essentially as described in [10,16]. Briefly, cells were mechanically lysed and the insoluble material was isolated by centrifugation across a sucrose cushion and further separated on a discontinuous sucrose gradient by ultracentrifugation (Fig 1A). Samples of the gradient were stained with DAPI and analysed microscopically. The pellet fraction contained the highest number of nuclei and little visible contaminants, such as kinetoplasts (disc-like network of circular DNA inside the single trypanosome mitochondrion, visible in the DAPI image) or flagella (visible in the brightfield image) (Fig 1B, right panels). This fraction will be referred to as NUC (nuclear fraction). In each nuclear purification experiment, one control sample was taken immediately after the mechanical lysis (whole cell lysate, WCL). As expected, whole cell lysates contained whole cell remnants with both nuclei and kinetoplasts (Fig 1B, left panels). Protein samples of the NUC and WCL fractions were analysed on a Coomassie-stained gel and by western blot. Histones were highly enriched in the nuclear fraction in comparison to whole cell lysates, while the amount of total proteins decreased (Fig 1C), in agreement with a successful enrichment of nuclei. All samples (4 x NUC and 4 x WCL) were subjected to label free quantitative (LFQ) mass spectrometry. 3447 protein groups were detected in at least 2 of the samples, corresponding to more than a third of all proteins encoded by the *T*. *brucei* genome [17] (S1A Table). The nuclear enrichment score (NES) of each protein group was determined as the ratio of LFQ intensities of the nuclear fraction divided by the LFQ intensity of WCL. To this end, LFQ values were transformed by log_2_ and the NES ranged from +7.7 to -9.2 (Fig 1D). The significance of the enrichment was determined by Welch’s t-test.

### Threshold definitions and GO-term analysis

Our aim was to produce a high-confident list of nuclear proteins, with few false positives. A comparison with experimentally validated non-nuclear compartments (described below) was subsequently used to evaluate the chosen thresholds. Initially, all proteins with an NES below 0.7 or a p-value above 0.05 were removed from the list. The threshold of 0.7 was chosen because it corresponded to a local minimum in the NES histogram (Fig 1D). This resulted in 760 candidate protein groups with nuclear localization. This cut-off is extremely stringent, as even some of the histones were excluded. In fact, a very high abundance of a protein reduces the difference between the nuclear LFQ and the total LFQ score. This was compensated in a second step by adding all proteins to the list with an NES above 0.7 if they were among the top 20% abundant proteins, independent of the p-value. This added only four more proteins to the list, but additional to Tb927.7.4180 and Tb927.11.2510 included two of the histones. Thus, the final list of nuclear protein candidates contains 764 protein groups (S1B Table); 239 of these are hypothetical proteins. For an initial quality control, we performed a Gene Ontology (GO) enrichment analysis with the tool provided by TriTrypDB [18]. We found 79 GO-terms for biological function more than 3-fold enriched within our 764 nuclear protein candidates in comparison to the whole genome (p-value <0.05) (S2 Table). These were almost exclusively GO-terms describing various processes of nuclear DNA and RNA metabolism, for example mRNA splicing, chromatin remodelling and transcription. There was only one exception, namely a GO term enrichment in long-chain fatty acid biosynthesis (GO:0042759 and GO:0001676), based on the presence of three fatty acid elongases (ELO1-3) in our nuclear proteome in comparison to four in the total genome. Fatty acid elongases are known to localise to the perinuclear region of the ER membrane in yeast [19] and this localisation appears conserved for the *T*. *brucei* enzymes, as shown by expressing an eYFP fusion of ELO3 [20]. Thus, the presence of fatty acid elongases in the nuclear proteome is likely caused by a co-purification of the nucleus-adjacent ER membrane.

### The nuclear proteome contains less than 2% false positives

**Fig 2:**
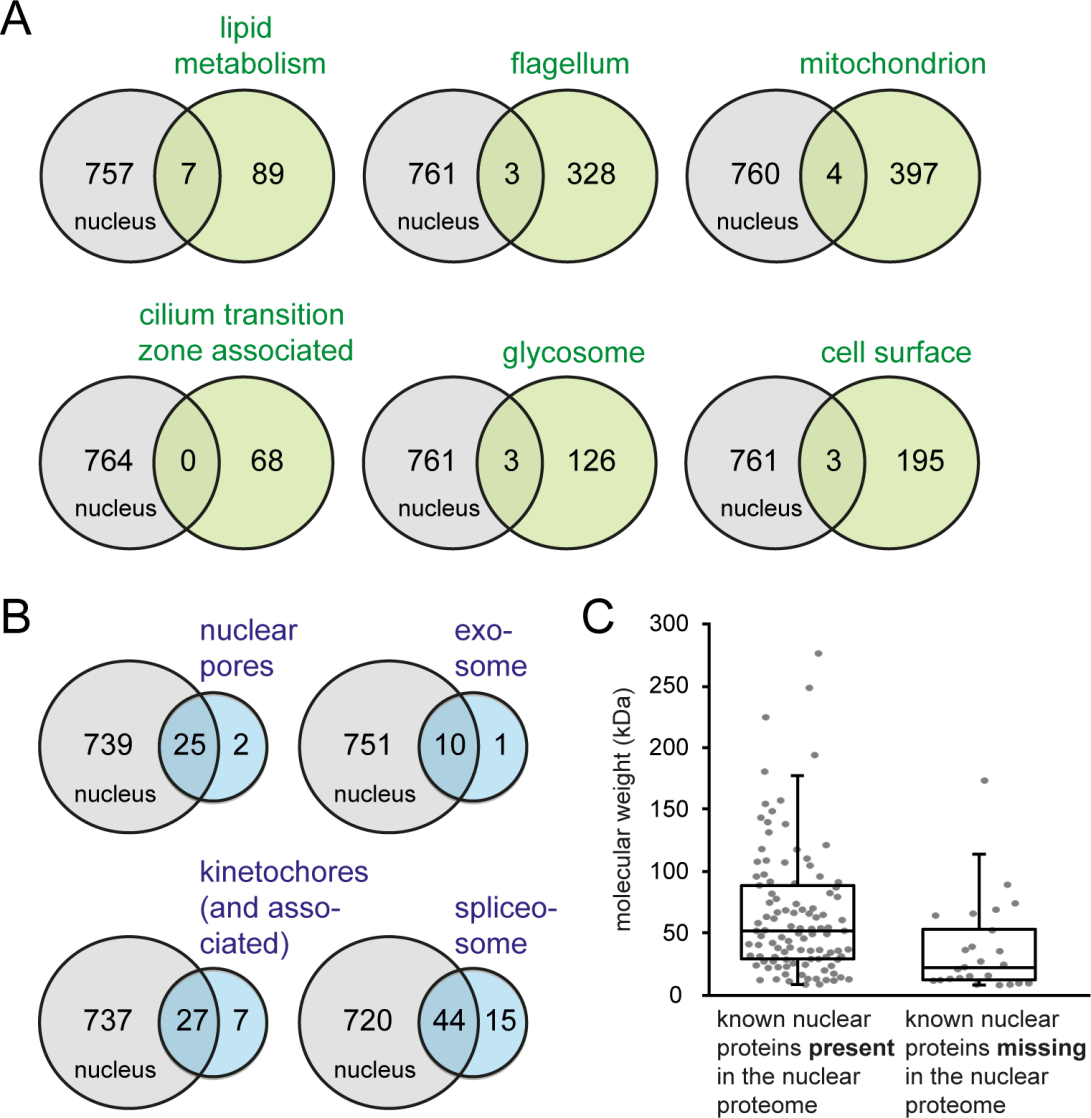
Comparison of the nuclear proteome with known nuclear and non-nuclear structures. **A)** The content of the nuclear proteome was compared with proteins involved in lipid metabolism, proteins of the flagellar proteome, proteins identified with high confidence in mitochondria, proteins tagged as part of the characterisation of the cilium transition zone, proteins of the glycosomal proteome and the cell surface proteome. The number of proteins that are present in both proteomes is shown in the overlap of the circles. **B)** The content of the nuclear proteome was compared with proteins of known nuclear structures: the nuclear pores, the exosome, the kinetochores and the spliceosome. The number of proteins that are present in both proteomes is shown in the overlap of the circles. **C)** The molecular weight of proteins from the known nuclear structures characterised in B is shown, for proteins that are present in the nuclear proteome (left) and for proteins that are absent from the nuclear proteome (right).

To estimate the number of non-nuclear proteins (false-positives) within our nuclear proteome, the proteome was compared with six experimentally characterised, non-nuclear structures/pathways: the lipid metabolism pathway [21], the flagellome [9], the mitochondrial proteome [11] proteins that associate with the cilium transition zone [22], the glycosome [14] and the cell surface [23] (Fig 2A).

There are 96 proteins described to be involved in *T*. *brucei* lipid metabolism based on homology to yeast enzymes and/or experimental characterisation [21]. Of these, seven are present in our nuclear proteome, including the three fatty acid elongases mentioned above (S3A Table). Many of the lipid metabolism proteins that are absent from our nuclear proteome localise to the ER, for example all enzymes involved in glycosylphosphatidylinositol (GPI) biosynthesis. This indicates that the contamination of our nuclear proteome with ER proteins seen in the GO-term enrichment analysis above is not a general phenomenon.

There are 331 proteins that were identified by mass spectrometry in purified flagella of *T*. *brucei* [9]. Of these, 16 are found in our nuclear proteome (S3B Table). However, for ten of them, nuclear localisation was demonstrated by the expression of GFP-fusion proteins [24,25] or by specific antibody staining [26]. Three of the remaining six proteins are clear homologues to proteins with nuclear localisation in other organisms, namely GLE2, Kre33 and ERB1. Thus, the actual number of possible flagellar proteins in our nuclear proteome is not higher than three (Tb927.5.940, Tb927.8.2290 and Tb927.3.5010).

The *T*. *brucei* mitochondrial proteome was determined from mitochondria enriched fractions [11]. The total mitochondrial proteome contains about 1000 proteins. For the comparison with the nuclear proteome, we focussed on the 401 proteins that were assigned to mitochondria with high confidence [11]. Of these 401 proteins, only four proteins are found in our nuclear proteome (S3C Table). They are likely false positives in our nuclear proteome as they are described by mitochondrial GO-terms and two of them are experimentally characterised, one is an RNA editing component [27] and another is found in the small subunit of the mitochondrial ribosome [28].

The proteome of the cilium transition zone was recently characterised [22]. As part of this study, 68 proteins were successfully localised by eYFP tagging to several different non-nuclear localisations (S3D Table). These included the cilium transition zone, the basal body, the pro-basal body, the flagellar pocket collar, the Inv-like compartment (a region distally adjacent to the transition zone), a longitudinal structure near the flagellum exit from the flagellar pocket, the flagellum, the Golgi and combinations of these localisations. Notably, there was no overlap between these 68 proteins and our nuclear proteome.

A proteome of the trypanosome glycosome was obtained by a combination of epitope tagged glycosome purification and SILAC labelling [14]. This study identified 129 glycosomal proteins with very high confidence. Accordingly, our nuclear proteome is contaminated with up to three glycosomal proteins (Tb927.5.2590, Tb927.8.920, Tb927.9.15260) (S3E Table).

The cell surface proteome of procyclic trypanosomes was obtained by mass spectrometry analysis of biotinylated surface proteins [12]. 198 unique protein groups, corresponding to 295 proteins, were identified (S3F Table). Of these, nine proteins are present in our nuclear proteome. Six of these have strong experimental evidence for nuclear localisation [29–31]. This leaves three proteins (all retrotransposon hot spot proteins, Tb927.1.120, Tb927.2.1330, Tb927.2.470) that could be false positives in our nuclear proteome; the absence of Tb927.1.120 from the nucleus was shown [29].

In summary, we have looked at 1162 unique proteins with non-nuclear localisation, excluding duplicates present in more than one proteome. Of these, 20 are present in our nuclear proteome and could therefore be false-positives, resulting in an estimated false positive rate of 1.7%.

### The nuclear proteome contains about 80% of the nuclear proteins

To estimate the comprehensiveness of the nuclear proteome, we compared it with the content of four well-characterised nuclear structures: the nuclear pores, the exosome, kinetochores and the spliceosome (Fig 2B).

Two studies have identified 27 structural components of the nuclear pores, excluding export factors [24,32] (S4A Table). The localisation of all proteins to a punctuate structure at the nuclear rim was confirmed by GFP tagging, with the exception of TbNup59 and TbNup62 which failed tagging [24,32]. Our nuclear proteome contains 25 of these 27 proteins; only TbNup75 and TbNup65 are absent.

The *T*. *brucei* exosome contains 11 known proteins [33–35] (S4B Table). Whether exosome localisation is entirely or only partially nuclear has been debated in the past, mainly based on contradictory results of cellular fractionation studies [35]. However, newer studies strongly support the view that the majority or all of the exosome is nuclear: all functions of the *T*. *brucei* exosome reported to date are nuclear [33,36–38] and eYFP tagging of the essential exosome component Rrp6 clearly showed dominant nuclear localisation mainly at the rim of the nucleolus with no or very little cytoplasmic fluorescence [38]. Of the 11 exosome proteins, ten were present in our nuclear proteome; only RRP41B was absent due to a slightly too high p-value.

Two recent studies aimed to describe the trypanosome kinetochores and identified 20 kinetoplast kinetochore proteins (KKT1-20), seven kinetoplast kinetochore interacting proteins (KKIP1-7)) and seven further nuclear proteins [30,39] (S4C Table). The nuclear localisation of all 34 proteins was confirmed by eYFP tagging [30,39]. Of these 34 proteins, 27 were present in our nuclear proteome. KKT1, KKT5, KKT10, KKT15, KKT16, KKIP5 and KKIP7 were absent.

The trypanosome spliceosome contains 59 known proteins, excluding all proteins that co-purify with spliceosomal components without a known function in splicing ([40] and references herein) (S4D Table). For most proteins, the localisation to the nucleus was not independently confirmed, but the trypanosome spliceosome is one of the best-characterised trypanosome structures: spliceosomal proteins of the different spliceosomal complexes were carefully identified by a combination of bioinformatics and tandem tag affinity purification with four different bait proteins, by many labs ([40] and references herein). Note that trypanosomes only have one heptameric Lsm complex, which is nuclear [41,42]. Of the known 59 spliceosomal proteins, 44 were present in our nuclear proteome. The 15 missing spliceosomal proteins included mostly small Lsm and Sm proteins.

To summarize, of the 131 proteins with known nuclear localisation, 106 are present in our nuclear proteome, corresponding to 80.9%. We therefore estimate the comprehensiveness of our nuclear proteome to about 80%. To note, very small proteins are preferentially absent: the average molecular weight of the nuclear proteins in our dataset (66 kDa) is significantly higher than the average molecular weight of the missing nuclear proteins (37.4 kDa) (result of unpaired, two-tailed students t-test = 0.01) (Fig 2C). Smaller proteins are more likely to be lost during the purification procedure by leaking out of the nucleus and result in fewer unique peptides detectable in the mass spectrometer.

### Identification of novel nuclear proteins

**Fig 3:**
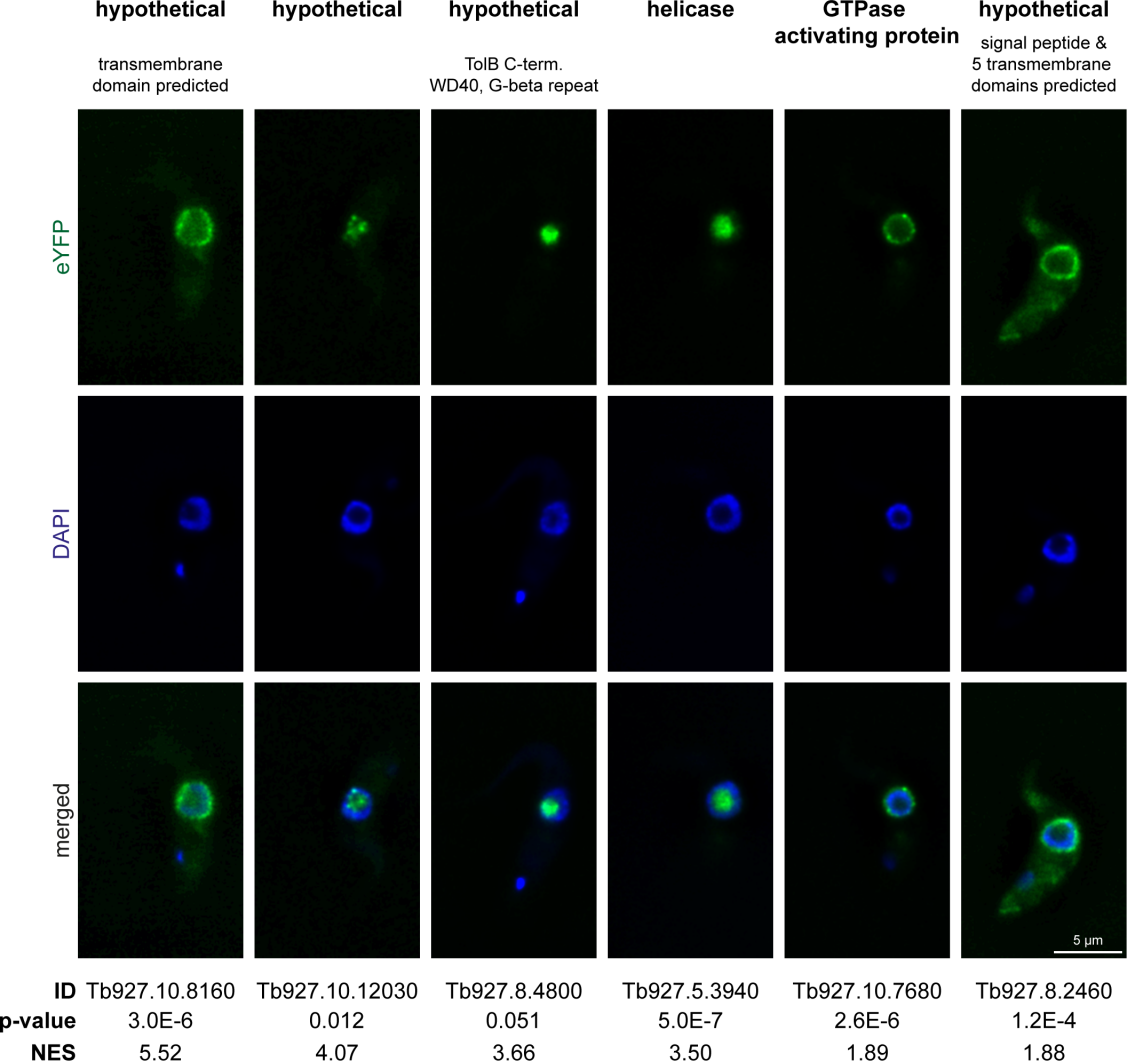
Validation of nuclear localisation by expressing eYFP fusion proteins. Six proteins of the nuclear proteome with previously unknown localisation were expressed as eYFP fusion proteins from their endogenous loci. Representative images (single plane images of deconvolved z-stacks) are shown. The DNA of the nucleus and the kinetoplast was stained with DAPI.

To investigate whether our proteome data set can be used to localize previously uncharacterized proteins, we expressed six proteins fused to eYFP from their endogenous loci [43]. These were four hypothetical proteins (including one with a p-value slightly above the threshold), one helicase and one GTPase activating protein with no available information about localisation (Fig 3). The NES values of these six proteins ranged from 1.8 to 5. Three proteins (Tb927.10.12030, Tb927.8.4800, Tb927.5.3940) were mainly in the nucleolus (visualised by the absence of DAPI staining) in one case (Tb927.10.12030) there were additional spots in the nucleoplasm. The GTPase activating protein (Tb927.10.7680) localised to a dot-like structure at the nuclear periphery, highly reminiscent of nuclear pores. The two remaining proteins (Tb927.10.8160 and Tb927.8.2460) localised to the nuclear rim, but the pattern was less spot-like and both proteins have predicted trans-membrane domains. This suggests localisation to the nuclear membrane, albeit a localisation to the nucleus-adjacent membrane of the ER cannot be excluded due to the limits of light microscopy.

### The motif KRxR is highly enriched in the nuclear proteins

A motif search with DREME [44] revealed three small peptide-motifs significantly enriched within or nuclear proteome: GSGKT, KRPR and KR[Q/E]R. The motif GSGKT is found in 31 proteins of the nuclear proteome (4.1%) and in 116 proteins (1.1%) of the total genome. The relevance of this enrichment remains unclear. The remaining two motifs are a sub-motif of the K[K/R]x[K/R] motif, which is the essential part of the monopartite classical nuclear localisation signal (NLS) [45]. The only known *T*. *brucei* protein with such a classical, experimentally characterised NLS is the LA protein; the sequence RGHKRSRE is both necessary and sufficient to mediate nuclear localisation [46]. The motif KRxR is present in 398 of the 764 proteins of the nuclear proteome at least once, thus in 52%. This represents a significant enrichment, compared to 17.7% of all trypanosome proteins (1810 of 10244 coding genes in the TREU927 strain). The position between the two arginine residues can be filled by any amino acid, except tryptophan. The most abundant amino acids at this position are arginine, proline, serine, glutamate and glutamic acid (S1 Fig). These results indicate that about half of all nuclear proteins could have a classical, monopartite NLS.

We propose that the KRxR motif can serve to predict nuclear localisation. It is present in 9 of the 25 known nuclear proteins that were absent from our nuclear proteome. Importantly, the KRxR motif could help to identify proteins that shuttle between the nucleus and the cytoplasm and have predominantly cytoplasmic localisation, as these are currently difficult to identify. The most prominent group of shuttling proteins, the group of ribosomal proteins, is not enriched in the KRxR motif and ribosomal proteins may thus use a different mechanism for nuclear entry. Notably, the absence of the KRxR motif does not exclude a protein from being nuclear. It is absent from almost half of all nuclear proteins and there are several other non classical nuclear localisation signals in trypanosomes [47].

## Conclusion

We provide a high-quality proteome of the *T*. *brucei* nucleus, which is about 80% complete and contains less than 2% non-nuclear proteins. The KRxR motif is highly enriched in nuclear proteins and could serve as a prediction tool for nuclear localisation. Nuclear proteins that are absent from the proteome are often of small size, and the 2% contaminants are enriched for proteins of the nucleus adjacent ER membrane. Note that the *T*. *brucei* nuclear proteome contains mainly proteins with exclusive nuclear localisation: proteins that shuttle between the cytoplasm and the nucleus with predominant cytoplasmic localisation are absent, as they are not enriched in the nucleus in comparison to the whole cell lysate. Recently, the proteome of the related kinetoplastid *T*. *cruzi* was determined and the number of nuclear proteins was in a similar range [48].

Our proteome data adds one more tool to the available sources for the study of trypanosome biology. Recently, TrypTag has started to systematically localise all *T*. *brucei* proteins [29]. We believe that our data are complementary to the current efforts of TrypTag. It may for example fill the gaps for the 10% of proteins that failed tagging or the fraction of the successfully tagged proteins with too low expression levels (Sam Dean, University of Oxford, UK, personal communication). Overall, our dataset will be useful to further untangle nuclear processes in trypanosomes.

## Supporting information

S1 Fig Frequency distribution of all amino acids at the position between the two arginine residues of the KRxR motif, for both the nuclear proteome and the total *T. brucei* proteome. (PDF)

S1 Table **A)** List of all proteins that were detected by mass spectrometry. **B)** List of the nuclear proteome.
(XLSX)

S2 Table GO-term analysis for biological function with the nuclear proteome. (XLSX)

S3 Table Lists of non-nuclear proteins, including overlaps with the nuclear proteome (estimation of false positives). (XLSX)

S4 Table Lists of nuclear proteins, including overlap with the nuclear proteome (estimation of missing proteins). (XLSX)
