# Supplementary material for: The nuclear proteome of Trypanosoma brucei
Source: PLoS One. 2017 Jul 20;12(7):e0181884. doi: 10.1371/journal.pone.0181884 (PMC5519215; doi:10.1371/journal.pone.0181884)
Supplement: S1 Fig — (PDF) [file pone.0181884.s001.pdf]

Figure S1

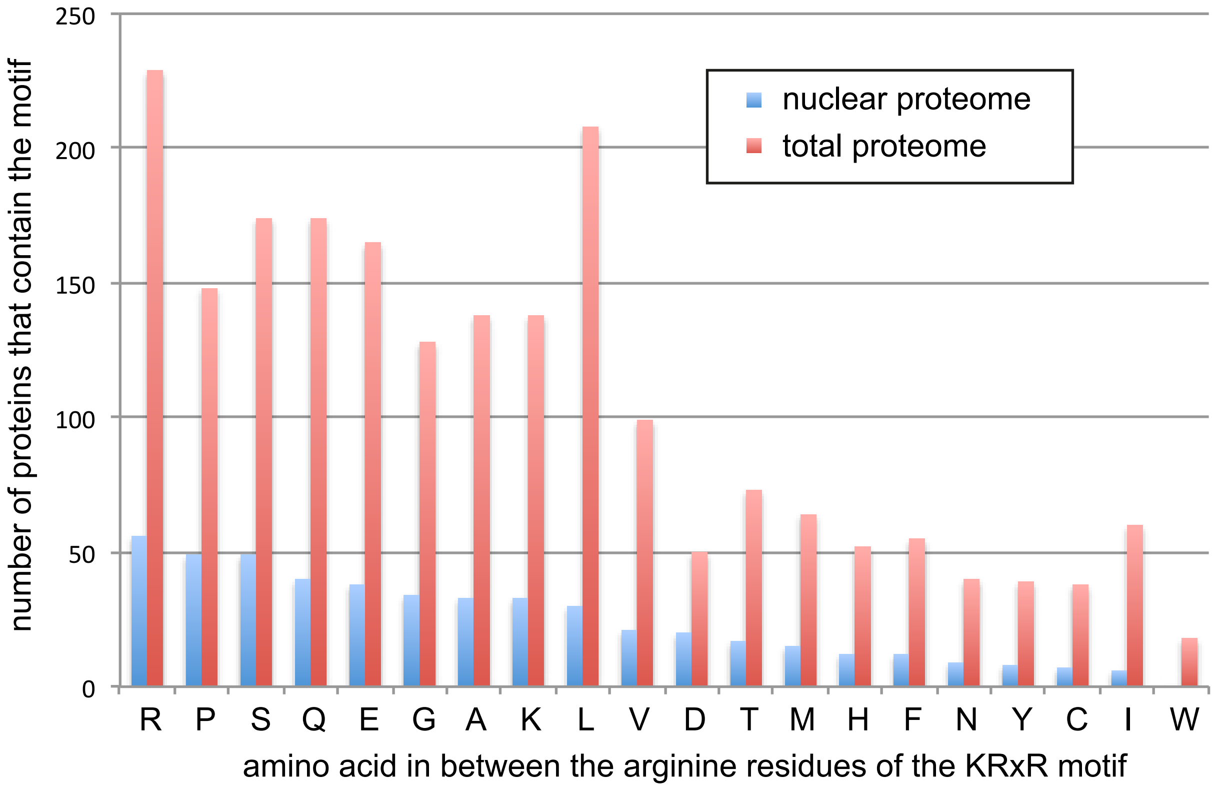

S1 Fig: The nuclear proteome and the total proteome of *T. brucei* Treu927 was searched for the KRxR motif with x being filled successively by all amino acids (x-axis). Note that the total number of proteins with the individual motifs is higher than the number of proteins that contain the KRxR motfif, because some proteins contain several mixed motifs.
